# Modeling titanium dioxide nanostructures for photocatalysis and photovoltaics

**DOI:** 10.1039/d2sc02872g

**Published:** 2022-07-25

**Authors:** Francesca Nunzi, Filippo De Angelis

**Affiliations:** Department of Chemistry, Biology and Biotechnology, University of Perugia Via Elce di Sotto 8 06123 Perugia Italy francesca.nunzi@unipg.it; Computational Laboratory for Hybrid/Organic Photovoltaics (CLHYO), Istituto CNR di Scienze e Tecnologie Chimiche (SCITEC-CNR) Via Elce di Sotto 8 06123 Perugia Italy; Department of Natural Sciences and Mathematics, College of Sciences and Human Studies, Prince Mohammad Bin Fahd University Khobar Dhahran 34754 Saudi Arabia

## Abstract

Heterogenous photocatalysis is regarded as a holy grail in relation to the energy and environmental issues with which our society is currently struggling. In this context, the characterization of titanium dioxide nanostructures and the relationships between structural/electronic parameters and chemical/physical–chemical properties is a primary target, whose achievement is in high demand. Theoretical simulations can strongly support experiments to reach this goal. While the bulk and surface properties of TiO_2_ materials are quite well understood, the field of nanostructures still presents a few unexplored areas. Here we consider possible approaches for the modeling of reduced and extended TiO_2_ nanostructures, and we review the main outcomes of the investigation of the structural, electronic, and optical properties of TiO_2_ nanoparticles and their relationships with the size, morphology, and shape of the particles. Further investigations are highly desired to fill the gaps still remaining and to allow improvements in the efficiencies of these materials for photocatalytic and photovoltaic applications.

## Abstract 2

The latest findings from theoretical investigations into TiO_2_ nanoparticles are reviewed, including both realistic models from a bottom-up approach (1–3 nm diameter) and cut from bulk models (>3 nm diameter) in a top-down approach.

## Graphical abstract

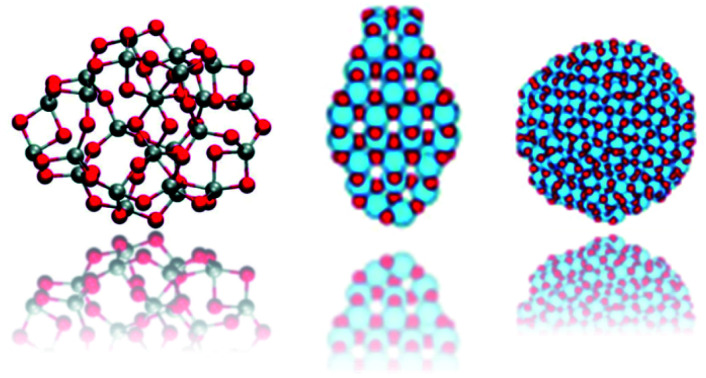

## 1. Introduction

The design of advanced semiconductors is of fundamental importance to accomplish the enhanced performances required in a variety of environmental and energy applications. In recent years, more and more serious air and water pollution, caused by the discharge of waste chemicals into the environment, strongly threaten human health. Moreover, the current massive production of energy with fossil fuels sets a double problem of fuel shortage and carbon dioxide accumulation, that can be overcome, or at least mitigated, by the exploitation of green and sustainable sources, such as solar energy. Remarkably, semiconductor photocatalysis^1,2^ can be regarded as the holy grail of solar energy conversion applications, that may fulfil fundamental tasks such as photocatalytic H_2_O splitting,^3^ photovoltaics,^4,5^ industrial waste degradation and biomass conversion.^6^

The majority of applications based on inorganic semiconductors involve nanostructured materials in the form of nanoparticles.^7,8^ The reduction in size down to the nanoscale (size range between 1 and 100 nm) introduces a significant improvement in the peculiar chemical and physical properties of a semiconductor with respect to the bulk solid.^9,10^ Semiconductor nanoparticles or nanostructures show a high surface area, involving a high number of reactive centres on the surface, that are able to promote the chance of higher efficiency in photocatalytic and photovoltaic processes.^8^ In addition, the opportunity to modify the morphology of the nanoparticles, by tuning not only the crystal polymorph, but also their shape and size, introduces further parameters to improve their electronic and optical properties.

The most widely used material in heterogeneous photocatalysis is titanium dioxide, TiO_2_.^11^ Among various TiO_2_ polymorphic phases, anatase has been found to be the most stable form for nanoparticle structures with approximate diameter below 20 nm, while rutile is the most thermodynamically stable bulk phase.^12^ A higher photocatalytic activity under ultraviolet light is usually encountered for TiO_2_ anatase nanoparticles compared to the rutile form, and its origin is probably related to multiple factors, such as morphology, size, defect chemistry and/or adsorbates.^11,13^

While the bulk and extended surfaces of TiO_2_ have been widely studied and characterized, both experimentally and theoretically, the investigation of TiO_2_ nanoparticles, being challenging from many points of view, is still partly open for discussion. Some experimental procedures for the synthesis of TiO_2_ nanoparticles with controlled shape and morphology have currently been established, but it is still difficult to discriminate between the effect of size/shape on photocatalytic activity and the effects introduced by the synthesis conditions. In this respect, quantum mechanical simulations can be very helpful in the investigation and rationalization of the individual parameters affecting the observed chemical and optical properties. Simulations based on electronic structure calculations, possibly from first principles, are strictly required to gain an accurate picture of the interplay between the structural and electronic factors governing the chemical and physical–chemical properties of TiO_2_ nanoparticles. Besides the employment of an accurate theoretical methodology, the choice of adequate TiO_2_ nanostructure models is essential to obtain results reliably related to the experimental evidence; this is a prerequisite to providing clues to optimize the experimental conditions, thus enabling the rationalization of the photocatalytic properties of the material.

Remarkably, the modelling of titania bulk crystals and clean surfaces involves the employment of theoretical methods based on periodic boundary conditions, that allows a significant restraint of the computational cost. On the other hand, realistic models of TiO_2_ nanoparticles necessarily involve a significant modelling effort, since the peculiar features characterizing the nanoparticles with respect to the bulk or clean surfaces arise from their finite size and shape, that give rise to numerous different undercoordinated sites, located at edges, corners or apical sites on the nanoparticles. In light of the importance of modeling and simulation strategies, in the present review the various possible approaches for modeling TiO_2_ nanostructures are encompassed with a sharp focus on nanoparticles, which are by far the most investigated structures. The reader is referred to the cited literature for an overview of the theoretical methods employed. The manuscript is organized as follows: the possible modelling strategies for TiO_2_ nanoparticles are illustrated, considering reduced (1–3 nm diameter) and larger (>3 nm diameter) sized particles (Section 2); afterwards the size dependence of energetic stability is unraveled, focusing on the non-crystalline to crystalline crossover size (Section 2.1). In Section 2.2 the dependence of the structural and electronic properties on nanoparticle size is reviewed, highlighting the regime size where these properties converge to those of the bulk materials. A focus on the optical and electronic gaps and the excitation energy is tackled, since these are fundamental parameters for the exploitation of TiO_2_ nanoparticles in photocatalysis and photovoltaics. In Section 2.3 the effect of the morphology on the electronic and optical properties is considered, analyzing faceted and spherical nanoparticles. Finally, the conclusions are outlined. More detailed information on the structural parameters of the reported structures can be found in the related papers.

## 2. Modelling strategies

The first step in the simulation of semiconductor nanoparticles is the development of adequate models, able to reproduce the main structural characteristics of the nanoparticles, while allowing an accurate calculation of their properties at an affordable computational cost. To reach this aim, two alternative modelling routes can be envisaged: (i) the “bottom-up” approach, where the model is assembled from atomic/molecular/cluster sized units; (ii) the “top-down” approach, where the model arises from a fine-tuned erosion of the bulk solid.^14^ The term “model” properly refers to a simplified representation of a real system. Nevertheless, in the case of nanoparticles of reduced size (1–3 nm diameter), an atom-by-atom reproduction of the entire system, avoiding any idealization, can eventually be adopted through the bottom-up procedure. Such an approach allows us to unravel, in the case of small nanoparticles, the variety of isomers differing in size, composition, morphology, and atomic and electronic structures. Because of the lack of bulk-like constraints, many different low-energy structures can arise. Among these, isomers resembling the bulk-like structure are generally quite unstable, while the isomers lowest in energy show an almost amorphous structure, markedly different from the bulk. The reduced size of the nanoparticles encompasses a discrete electronic structure, that strongly characterizes the nanoparticles with respect to the bulk and provides peculiar chemical, optical and magnetic properties. Remarkably, analysis of the structural and electronic properties of the nanoparticles may suggest not only the size-dependent limit of the bulk-properties, but also information about the initial stages of aggregation and nucleation of larger nanoparticles and bulk polymorphs.

For more extended nanoparticles, with diameters larger than 3 nm, the exploitation of a model procedure to represent the real system is mandatory and it involves the choice of the overall shape, exposed facets and surface area of the nanoparticles, generally through a top-down approach. A common procedure is based on the Wulff construction,^15^ that correlates the equilibrium shape of a crystal with the minimum of the total surface Gibbs energy. In the case of oxide nanoparticles, because of the atomic nature of the system and of the introduction of the surface energies and surface areas of various crystal faces, the Wulff construction leads to the definition of a closed polyhedron, reproducing the equilibrium form of the nanoparticles. The identified nanoparticle shape may undergo a further refinement on the basis of more elaborate methods, also including edge and corner energies, surface tension and bulk elastic energy. In the case of TiO_2_ anatase nanocrystals, the Wulff construction suggests a truncated bipyramidal shape, massively exposing (nearly 94%) the thermodynamically most stable (101) surfaces and some degree of (001) truncation of the apices. This prediction is generally confirmed by experiments, even if peculiar experimental conditions may enforce the synthesis of nanoparticles where the percentage of (001) facets extends to roughly 47%.^16^ In addition, more rounded nanoparticles can also be obtained,^17^ eventually controlling the hydroxylation of the environment, even if anhydrous spherical nanoparticles are sometimes reported. Table 1 provides a resumé of the main features of the discussed TiO_2_ models.

**Table 1:** TiO_2_ modelling approaches and main characteristics

“Bottom-up” (from atomic/molecular/cluster scale)	“Top-down” (cut from the bulk)
Particles 1–3 nm in size	Particles with diameters > 3 nm
Realistic models (no idealization)	Modelling is based on the overall shape, exposed facets, and surface area
Several low-energy isomers, structurally different from the bulk	3 types of models, mostly reproducing the bulk crystal structure: (i) truncated octahedral shape [(101) and (100) facets]; (ii) bipyramidal shape [exclusively (101) facets]; (iii) almost spherical shape

While, in general, a reduction in nanoparticle size is expected to enhance the photoactivity, because of the higher surface area ratio, it also enforces a disruption of the crystalline order for nanoparticles with a diameter below ∼5 nm and eventually of the electronic properties peculiar to bulk-like anatase samples, related to high photocatalytic performance. Moreover, the decreasing size of the nanoparticle involves an increase in quantum confinement,^18–21^ that is expected to increase the optical energy gap, thus promoting a reduction in photoactivity. Accordingly, it is fundamental to carefully analyse the dependence of the photochemical properties on the nanoparticle size to clarify which of the various concurrent effects prevails. The morphology and shape of nanoparticles can also play key roles in modulation of photoactivity.

### 2.1 Size-dependent energetic stability

**Fig. 1:**
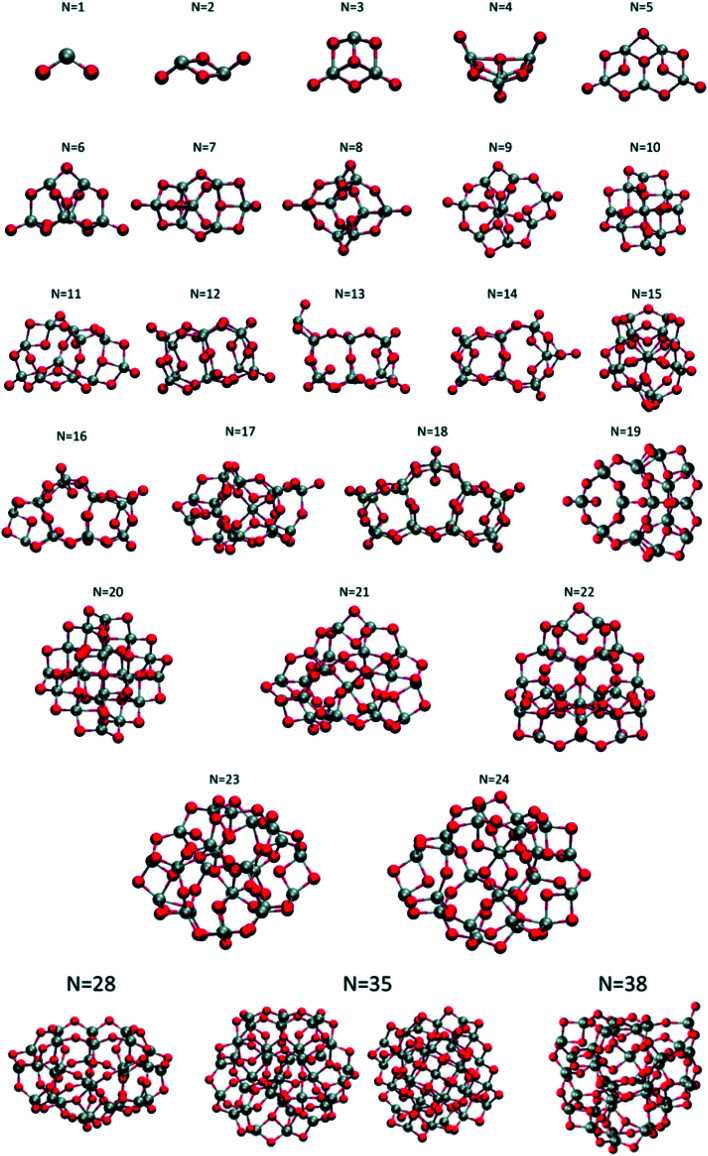
Atomic structures of the low-energy bottom-up (TiO_2_)_*n*_ nanocrystals investigated in ref. 20. For *N* = 35, the structure on the right was obtained by data mining from a tetrahedral (CeO_2_)_35_ nanoparticle in ref. 22. Titanium, red; oxygen, grey. Reproduced from ref. 20 with permission from the Royal Society of Chemistry.

Lamiel-Garcia and co-workers investigated by first principles calculations the structure of several (TiO_2_)_*n*_ nanoparticles, with *n* ranging from 1 to 84, highlighting the size dependence of the energetic stability.^20^ The authors distinguished between titania nanoclusters and nanocrystals, referring, respectively, to the absence or presence of anatase crystalline order. By employing the bottom-up procedure and starting from a TiO_2_ monomer, the (TiO_2_)_*n*_ nanoclusters need to attain a certain size before a bulk-like structure, characterized by a regular crystal ordering, becomes thermodynamically favoured over an amorphous one. In this regard, the structural crossover between nanocluster and nanocrystal nanoparticles can be seen as a size-dependent non-crystalline (NC) to crystalline (C) crossover. Specifically, an NC size range refers to nanoparticles lacking full crystallinity, while a C size range refers to nanoparticles cut from a bulk crystal through a top-down approach and exhibiting almost unaltered atomic ordering upon structural relaxation, with bond length variation within 20%. The authors provide an estimate of the NC ↔ C crossover size regime for TiO_2_, analysing the energetic stability of nanoclusters and nanocrystals derived from bottom-up and top-down approaches, respectively. Remarkably, the NC ↔ C crossover will not generally point to the most thermodynamically stable bulk crystalline phase of the material, but rather to a polymorph, which is metastable in the bulk. Global optimization and data mining on bottom-up-derived (TiO_2_)_*n*_ clusters with *n* = 1–38 (*i.e.* up to 114 atoms) lead to the definition of low-energy structures lacking anatase crystal order (see Fig. 1). By increasing *n*, the number of terminal dangling oxygen Ti–O defects decreases and clusters with *n* > 18 are either fully coordinated (*i.e.*, zero terminal defects) or have at most one Ti–O defect. For the larger bottom-up-derived optimized clusters (*n* = 28, 35, 38; see Fig. 1) the energy gain with respect to the corresponding anatase nanocrystals is very high (7.9–9.9 eV). These results suggest the inadequacy of metastable anatase nanocrystal structures within this size range to model more extended bulk-like TiO_2_ nanoparticles.

**Fig. 2:**
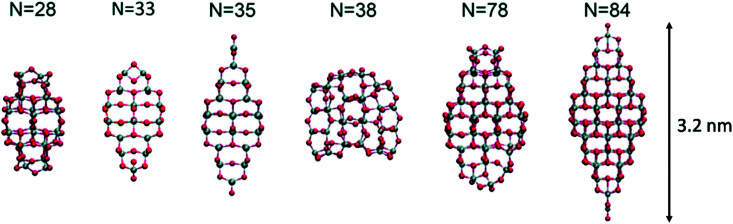
Atomic structures of the top-down (TiO_2_)_*n*_ nanocrystals investigated in ref. 20. The scale bar on the right strictly refers to the larger model. Titanium, red; oxygen, grey. Reproduced from ref. 20 with permission from the Royal Society of Chemistry.

**Fig. 3:**
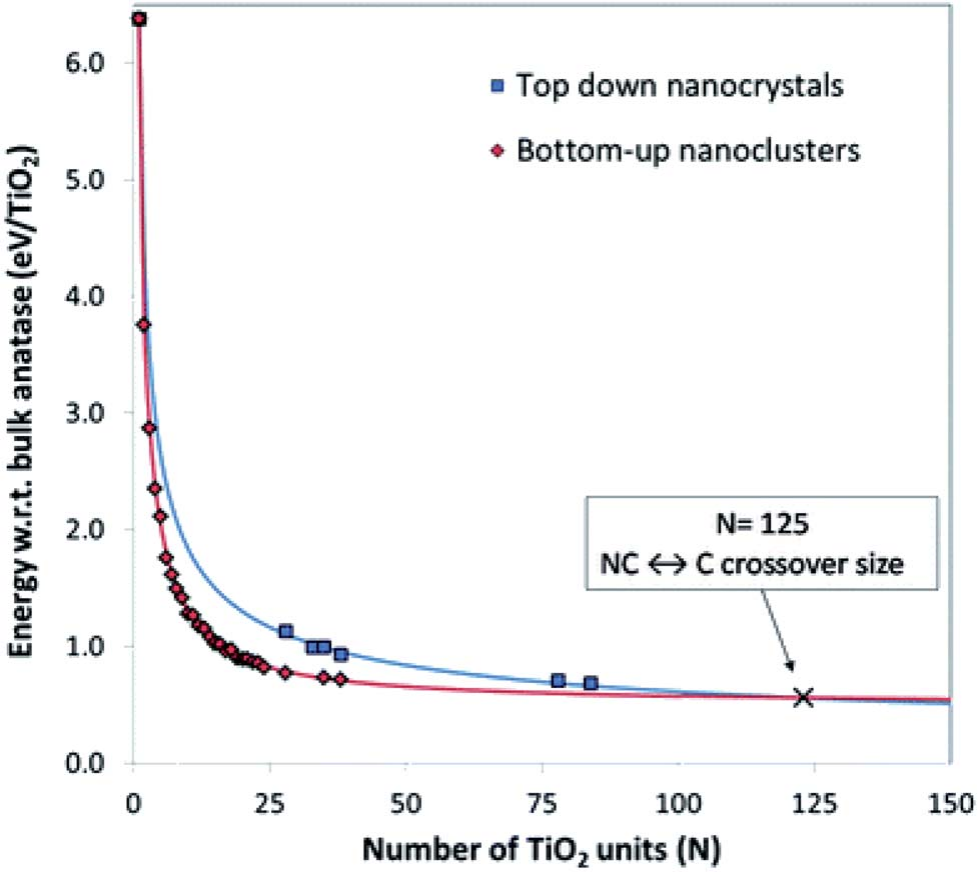
Energies of bottom-up and top-down (TiO_2_)_*n*_ nanoclusters with the corresponding fitting lines. The NC ↔ C crossover size is indicated by the cross at *n* = 125 (*i.e.*, 375 atoms). Reproduced from ref. 20 with permission from the Royal Society of Chemistry.

Lamiel-Garcia and co-workers^20^ also considered larger (TiO_2_)_*n*_ nanocrystals with *n* = 33–84 (*i.e.* 84–252 atoms) obtained from a top-down approach, by suitable cutting from the bulk anatase crystal. After structural relaxation, these nanocrystals adequately retain the bulk-like atomic structure, adopting various morphologies (truncated bipyramidal, bipyramidal or spherical), as reported in Fig. 2. By employing nanoparticle and nanocrystal data sets and fitting them with a generalised expansion of a top-down-derived energy expression for nanoparticles, the NC ↔ C crossover size at which anatase nanocrystals become more energetically stable than nanoclusters is estimated for *n* = 125 (*i.e.* 375 atoms), corresponding to 2–3 nm diameter nanoparticles, as reported in Fig. 3. This result is in agreement with the experimental evidence showing below such a size the dominance of amorphous spherical titania nanoparticles *versus* faceted anatase nanocrystals.^23^

### 2.2 Structural and electronic properties

**Fig. 4:**
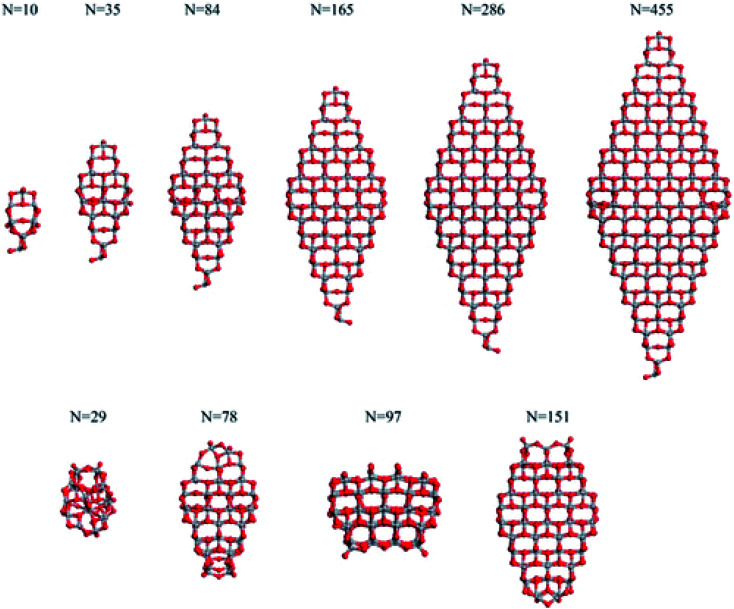
The octahedral (top) and cuboctahedral (bottom) (TiO_2_)_*n*_ nanoparticles investigated in ref. 21. Reprinted with permission from O. Lamiel-Garcia, K. C. Ko, J. Y. Lee, S. T. Bromley and F. Illas, *J. Chem. Theory Comput.*, 2017, **13**, 1785–1793. Copyright 2017 American Chemical Society.

Analysis of the dependance of the structural and electronic properties of TiO_2_ nanoparticles with increasing size may suggest the range of particle diameters where such properties converge linearly with those of the bulk material. Such an approach would allow the properties of extended nanoparticles to be foreseen on the basis of those computed for the smaller counterparts. In this perspective, Lamiel-Garcia *et al.*^21^ considered ten different stoichiometric (TiO_2_)_*n*_ anatase nanoparticles (*n* = 10–455, closely corresponding to 1–6 nm diameter) to investigate the structural and electronic structures and their dependance on both the size and shape of the nanoparticles by DFT methods, comparing the results from various density functionals. On the basis of the Wulff construction approach, the authors considered two kinds of bipyramidal models, one with octahedral symmetry, exposing exclusively (101) facets, and one with a truncated octahedral shape, exposing both (101) and (001) surfaces (see Fig. 4). Such particles extend up to 6 nm in height, thus resembling, both in size and shape, the experimentally synthesized particles.^24^

**Fig. 5:**
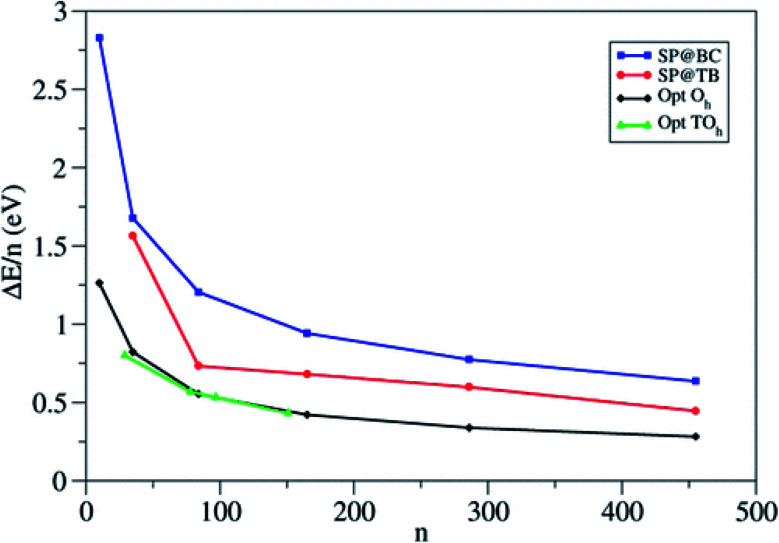
Energy per TiO_2_ unit relative to anatase (Δ*E*/*n*) for the octahedral (O_h_) and truncated octahedral (TO_h_) (TiO_2_)_*n*_ nanoparticles investigated in ref. 21. For comparison, the authors included the values corresponding to the unrelaxed structure cut from the bulk and to the tight-binding relaxed structures from Barnard *et al.*^25^ Blue squares: single-point calculations using the bulk-cut structures (SP@BC); red dots: single-point calculations using the tight-binding relaxed structures (SP@TB); black diamonds: optimized O_h_ structures (Opt O_h_); green triangles: optimized TO_h_ structures (Opt TO_h_). Reprinted with permission from O. Lamiel-Garcia, K. C. Ko, J. Y. Lee, S. T. Bromley and F. Illas, *J. Chem. Theory Comput.*, 2017, **13**, 1785–1793. Copyright 2017 American Chemical Society.

The authors compare the relative stability of PBE-optimized (TiO_2_)_*n*_ nanoparticles *vs.* unrelaxed bulk-cut anatase structures by computing the corresponding energy difference per TiO_2_ unit (Δ*E*/*n*) (see Fig. 5). For small particles, Δ*E*/*n* drops rapidly with increasing *n*, with a decrease of 0.47 eV per unit going from *n* = 10 to 35, while for large particles it decreases smoothly (only 0.06 eV per unit going from *n* = 286 to 455). Octahedral and truncated octahedral models show almost the same trend, suggesting that the size effect prevails over the morphological one. Fig. 5 also shows that the relaxation energy of the optimized structures with respect to the bulk cut is very large for smaller particles (2 eV for *n* = 10), thus pointing to strong structural reorganization with respect to the bulk, while it asymptotically decreases for larger ones (only 0.7 eV for *n* = 455). The computed Ti–O bond distances for *n* > 35 differ by less than 0.05 Å from the bulk value (1.95 Å), while the average Ti and O coordination number only slowly converges to the bulk value, probably due to the presence of undercoordinated sites on the particle surface. These results suggest that larger nanoparticles (*n* > 35) show a relatively unperturbed anatase structure, with defined (100) and (001) facets.

A comparison of the electronic structure of the nanoparticles with respect to bulk TiO_2_ material can be obtained by comparing the values of the optical and electronic gaps.^26–28^ Since the two terms have often been confused, it is worth specifying the definitions of each. The electronic (or fundamental, *E*_gap_) gap involves charge states of cationic (free extra hole, h^+^) or anionic (free extra electron, e^−^) character and corresponds to the difference between the ionization potential and the electron affinity. It is measured by (direct and inverse) photoemission spectroscopy, while computationally it requires a comparison between the total energy of the *N*-electron ground state and that of the *N* + 1/*N* − 1 electron state to determine the ionization potential/electron affinity.^28^ On the other hand, the optical gap (*O*_gap_) is gained by a photoexcitation process and corresponds to the energy of the lowest electronic transition accessible *via* absorption of a single photon, that generates an excited electron–hole (e^−^–h^+^) pair, a so-called “exciton pair”. The exciton is a bound state where the excited electron and hole are electrostatically attracted. The optical gap is experimentally measurable from optical spectroscopy, while its computation involves vertical excitation from the ground to the first excited state (usually singlet rather than triplet), by considering the difference between the energy of the excited state in the ground-state geometry and that of the ground state, therefore requiring sophisticated methods, such as configuration interaction, TD-DFT or GW many-body quasi-particle. However, the optical gap can be safely approximated by the difference in the DFT Kohn–Sham energy levels of the LUMO and HOMO, especially useful in the case of extended systems involving huge computational effort. It can be proved that the HOMO–LUMO gap is a better approximation of the optical rather than the fundamental gap.^21,28–30^

The difference between the electronic and optical gaps is the exciton binding energy, corresponding to the energy needed to separate the electron and hole, converting them into free charge carriers. A large exciton binding energy suggests a strong electron–hole pair, that can lead to rapid charge recombination, inhibiting the photochemical reactions on TiO_2_ nanoparticles. In relatively small finite systems, the optical gap is smaller than the electronic gap, because of the electrostatic stabilization of the electron–hole pair interaction in the excited state. In the bulk and surface materials, because of crystalline periodicity, the molecular energy levels are replaced with electronic bands. The electronic gap is properly identified as the band gap, corresponding to the difference between the bottom of the conduction band and the top of the valence band. The optical gap still refers to the energy of the lowest optical transition. Conversely to nanoparticles, in the case of extended systems the difference between the two gaps, and therefore the exciton binding energy, becomes negligible (generally of the order of a few meV), since the electronic structure is scarcely affected by the addition/removal of one electron to/from the delocalized state.^26,31,32^

Accordingly, calculation of the exciton binding energy values provides an estimation of the diversity in the electronic structure of nanoparticles of increasing size with respect to bulk materials. Such an approach has been considered by Lamiel-Garcia *et al.*,^21^ who, however, introduced an approximation into the calculation of the optical gap because of the considerable size of the investigated nanoparticles, by simply considering the difference of the HOMO–LUMO Kohn–Sham energies, as validated in previous work.^28^ Because of the well-known dependency of the Kohn–Sham energy levels on the DFT functional, various functionals with different HF percentages have been considered. The calculations prove that the optical gap depends slightly on the nanoparticle size, conversely to the electronic gap, rapidly decreasing and asymptotically converging with size to the bulk anatase value. The effect of the morphology on the optical and electronic gaps is secondary. Accordingly, the exciton binding energy exhibits a decreasing trend with size, moving from energy values of 3 eV to less than 1 eV for the largest model with the selected PBEx functional,^33,34^ where the HF percentage has been calibrated to 12.5% to reproduce the experimental value for bulk TiO_2_ (3.20 eV).^35,36^ In addition, starting from the computed values, the authors considered an extrapolation procedure to define the size at which the TiO_2_ nanoparticles will exhibit a bulk-like electronic structure. They concluded with an ∼20 nm apical diameter for octahedral nanoparticles exhibiting the most stable (101) surfaces, in agreement with previous indications derived from smaller nanoparticles.^28^

As stated above, by carefully tuning the synthetic procedures, varying the TiO_2_ precursors and surfactants,^37–40^ nanoparticles with different morphologies can be obtained. In agreement with the Wulff construction,^15^ bipyramidal and truncated octahedral crystalline anatase TiO_2_ nanoparticles with (101) and (101)/(001) surfaces are commonly produced,^41^ but roughly spherical nanoparticles have also been produced experimentally.^17^ Spherical TiO_2_ nanoparticles, because of their high curvature profile, involving numerous undercoordinated sites, usually present higher binding properties and higher chemical activity with respect to faceted nanoparticles. Nevertheless, the experimental characterization of TiO_2_ nanoparticles with a highly curved surface is extremely difficult, so that high-level electronic structure calculations are fundamental to gain insights into the structural and optical properties of these peculiar nanostructures.

### 2.3 Shape-dependent electronic properties: rounded *vs.* octahedral nanoparticles

**Fig. 6:**
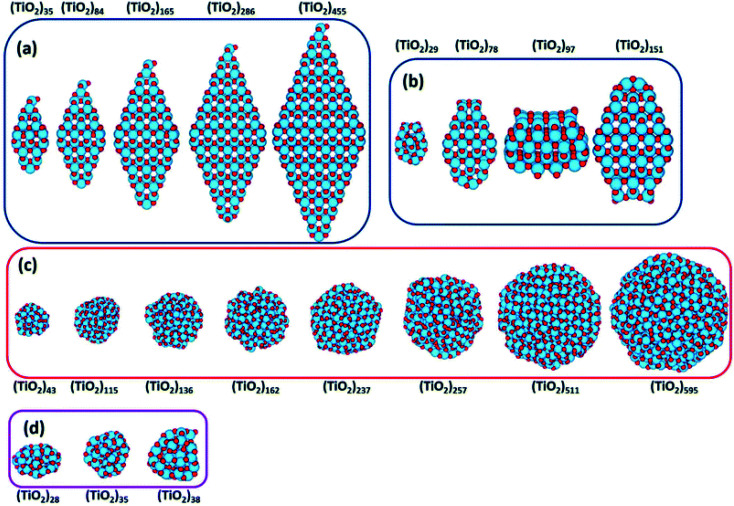
(TiO_2_)_*n*_ nanoparticles (*n* = 28–595) with (a) bipyramidal, (b) cuboctahedral, and (c) spherical morphologies, and (d) quasi-spherically globally optimised structures investigated in ref. 42. Red/blue spheres correspond to O/Ti atoms. Reproduced from ref. 42 with permission from the Royal Society of Chemistry.

Recently, Morales-Garcia *et al.*^42^ investigated more extended (TiO_2_)_n_ nanoparticles, with *n* up to 595 and a diameter up to 3.5 nm, unravelling not only the effect of size, but also of morphology on optical properties. Following a top-down approach, they considered both faceted (bipyramidal and truncated octahedral) and spherical stoichiometric (TiO_2_)_*n*_ nanoparticles; see Fig. 6. Geometrical optimizations were followed by thermal annealing. Experimentally, thermal annealing is often used to reduce the number of defects and to induce changes in the crystallinity and morphology of TiO_2_ nanoparticles.^43,44^ Molecular dynamic simulations followed by geometrical optimizations allow us to simulate the experimental annealing effect.

**Fig. 7:**
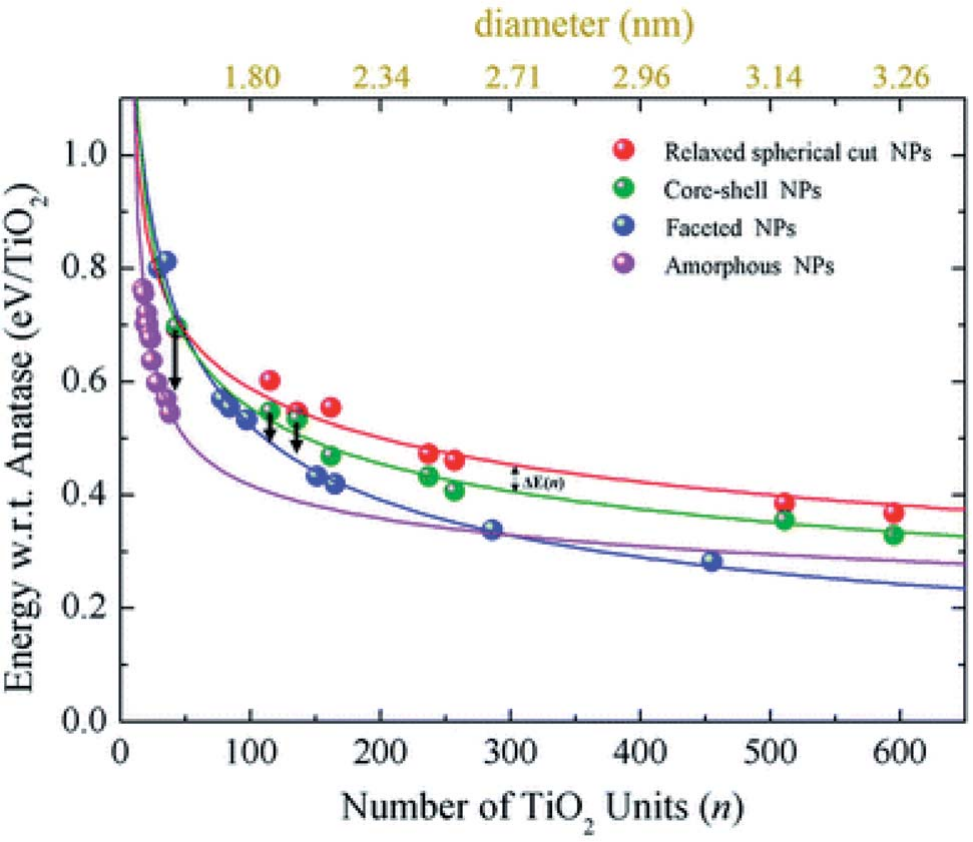
The energy per TiO_2_ unit with respect to bulk anatase (set to zero) of faceted (blue), spherical cut (red), annealed core–shell spherical (green), and amorphous (magenta) (TiO_2_)_*n*_ nanoparticles as a function of n. An estimation of the diameter is reported *via* the upper *x*-axis. The black arrows indicate the energetic stabilisation of the (TiO_2_)_43_, (TiO_2_)_115_, and (TiO_2_)_136_ core–shell nanoparticles upon annealing to nanoparticles with fully amorphized structures. Reproduced from ref. 42 with permission from the Royal Society of Chemistry.

Relaxed faceted TiO_2_ nanoparticles are computed to be energetically more stable than spherical structures and the energy difference grows approximately linearly with the number of TiO_2_ units; thus rationalizing the dominance of faceted *versus* spherical particles at larger size. However, simulated annealing processes lead to a consistent increase in the energy stability of spherical particles, while leaving almost unaffected both the structure and the energy of the faceted models. Analysis of the geometrical parameters points out that spherical TiO_2_ nanoparticles, conversely to faceted ones, undergo a significant structural rearrangement, with an increase in the percentage of undercoordinated, Ti_4c_ and O_2c_, centres upon annealing. The all-electron calculations point out that the non-annealed spherical nanoparticles are always metastable with respect to both annealed spherical and faceted nanoparticles. However, the size of the nanoparticle is a key feature for both shape and morphology. In fact, for the larger investigated (TiO_2_)_*n*_ models (diameter > 3 nm, *i.e. n* > 400), the crystalline faceted are the most stable species, while for smaller ones, (diameter < 1.8 nm. *i.e. n* < 100), quasi-spherical amorphous structures are computed to be lower in energy. For intermediate-size particles (2–3 diameter, *i.e.*, *n* = 150–400), a spherical shape with a bulk crystalline order limited to the core and an amorphous shell (core–shell nanoparticles) is favoured upon annealing (see Fig. 7). Indeed, the annealing process is expected to mainly affect the surface atoms, that represent a major fraction for smaller particles, so that the energy stabilization effect upon annealing is expected to be greater at small particle size.

**Fig. 8:**
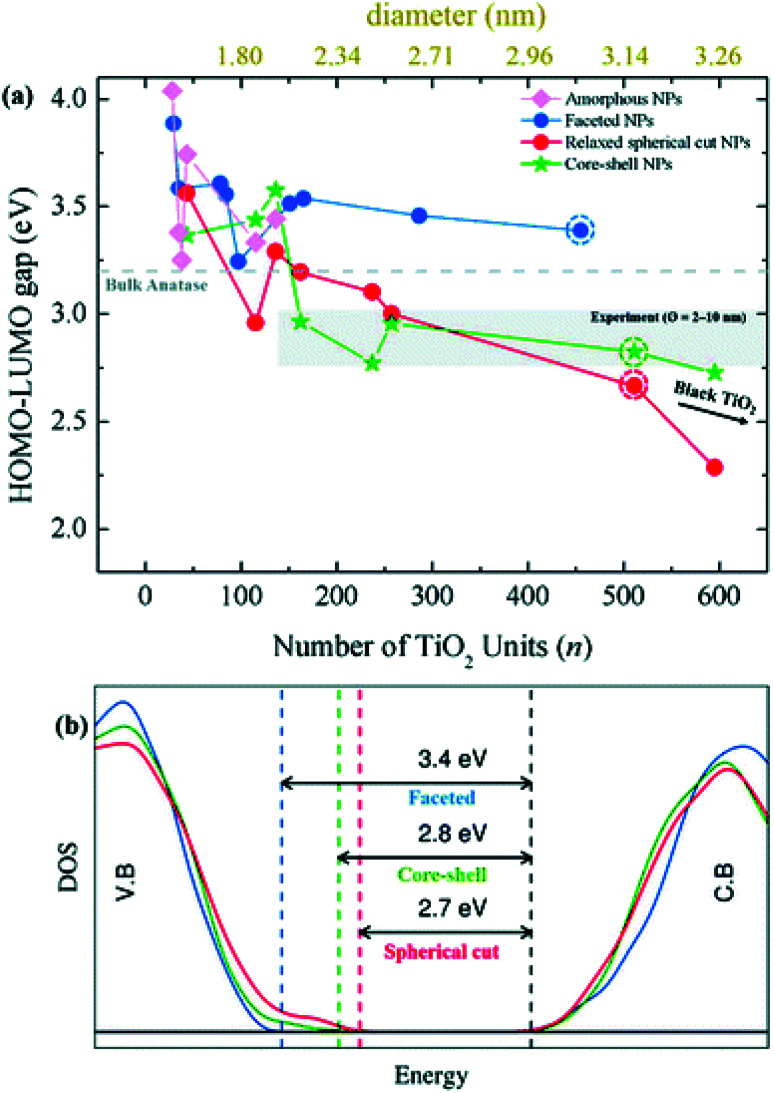
(a) Optical gap estimated as the HOMO–LUMO difference for faceted (blue), spherical cut (red), annealed core–shell spherical (green), and amorphous (magenta) (TiO_2_)_*n*_ nanoparticles as a function of *n*. (b) The density of state (DOS) of faceted (TiO_2_)_455_ and relaxed direct cut and annealed spherical (TiO_2_)_511_ nanoparticles—corresponding to the circled data points in (a). Reproduced from ref. 42 with permission from the Royal Society of Chemistry.

Besides the energetic stability, the authors considered the variation in optical gap, as predicted by the Khon–Sham orbital energies from DFT calculations, with respect to nanoparticle size and morphology, since it is a parameter strictly related to the performances of the material in photocatalysis. By employing the PBE functional with an increased Hartree–Fock exchange percentage (12.5%, labelled PBEx), the authors revealed that nanoparticles with diameter less than 2.0 nm have an optical energy gap higher (at most 1 eV) than the reference bulk anatase (3.2 eV), independent of the morphology [see Fig. 8(a)]. A different picture is returned in the higher size regime (>2 nm), where the thermodynamically more stable anatase-faceted nanoparticles still show an optical energy gap much higher than the bulk, while the spherical nanoparticles, both relaxed and annealed, have an optical energy gap which is significantly lower. Interestingly, in the 2–10 nm size range the computed values, especially for the annealed core–shell spherical nanoparticles, are in good agreement with the experimental values measured for annealed nanoparticles.^45^ The reduction in the energy gap, a particularly appealing feature for photocatalysis applications, is rationalized on the basis of a disorder-induced broadening of the valence band edge in spherical nanoparticles, as also observed in larger TiO_2_ black nanoparticles.^46,47^ The analysis of the density of states (DOS) in core–shell nanoparticles, reported in Fig. 8(b), shows that the undercoordinated Ti_4c_ and O_2c_ sites, that peculiarly characterize the spherical *vs.* faceted structures, significantly contribute to the broadening of the valence band. It is worth noting that the energy gap decreasing with increasing size can also be related to a decrease in quantum confinement.^18–21^ Remarkably, the investigation of Morales-Garcia *et al.*^42^ highlights that energy gap engineering can be induced in TiO_2_ nanoparticles by tuning the disorder in quasi-spherical nanoparticle structures, without altering the stoichiometry or introducing dopant agents.

**Fig. 9:**
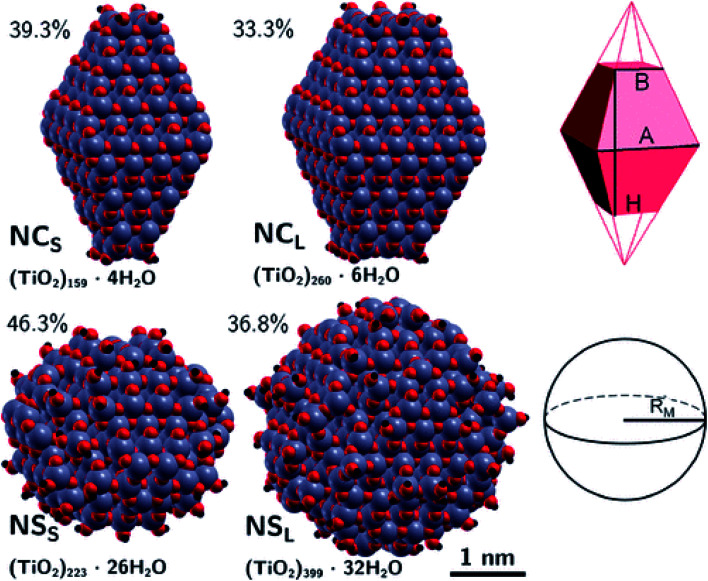
Top: faceted TiO_2_ NPs and a corresponding schematic representation of the Wulff-shape decahedron. Bottom: spherical TiO_2_ nanoparticles and a corresponding schematic representation of the sphere. The surface-to-bulk percentage ratio and stoichiometry are also shown. Reprinted with permission from G. Fazio, L. Ferrighi and C. Di Valentin, *J. Phys. Chem. C*, 2015, **119**, 20735–20746. Copyright 2015 American Chemical Society.

The differences between faceted and spherical anatase TiO_2_ are accurately marked in the work of Fazio *et al.*,^48^ by using DFT calculations to analyze four models up to 3 nm in size, two with a bipyramidal truncated shape [(TiO_2_)_159_·4H_2_O and (TiO_2_)_260_·6H_2_O, respectively denoted NC_S_ and NC_L_, that is, small and large nanocrystals] and two with a spherical shape [(TiO_2_)_223_·26H_2_O and (TiO_2_)_399_·32H_2_O, respectively denoted NS_S_ and NS_L_, that is, small and large nanospheres]; see Fig. 9. The stoichiometry of the models obtained by a top-down approach from optimized bulk anatase structures is guaranteed by the removal of dangling atoms and saturation with OH groups or H atoms, respectively, for Ti_4c_ or O_2c_ sites, as derived from water dissociation of TiO_2_ nanoparticles. The size of the particles has been compared in terms of the equivalent diameter derived from the Connolly volume,^49,50^ showing that the average size is roughly 2–3 nm, consistent with real small nanocrystallites.^51^ Moreover the size of the NC_L_ and NS_S_ models is almost comparable, both in terms of number of atoms and equivalent diameter, so that potential differences in the electronic structure can be attributed to the difference in shape. The NC models are characterized by a higher percentage of Ti_5c_ sites, localized on the edge and (101) lateral surfaces, and by a lower percentage of undercoordinated (<5c) Ti sites with respect to the NS models. The arrangement of the O_3c_ and O_2c_ sites is almost unaffected by the nanoparticle shape.

Structural distortions of the nanoparticle Ti–O bonds with respect to bulk have been evaluated by simulation of X-ray absorption fine structure (EXAFS), pointing out that the peak corresponding to the first coordination sphere is broader for NS than for NC models, thus confirming the presence of a wider variety of undercoordinated sites and a more disordered surface in spherical than in faceted particles.

The extent of the nanoparticle surfaces has been compared in terms of Connolly surfaces, showing that an NC has a larger surface area than an NS of comparable size, while the surface-to-bulk ratios are greater for NS than for NC particles and they decrease with increasing size. Higher surface energies have been computed for nanoparticles with respect to the regular (101) surface, especially for NSs. All these structural features concur to suggest higher reactivity of NSs than NCs.

**Fig. 10:**
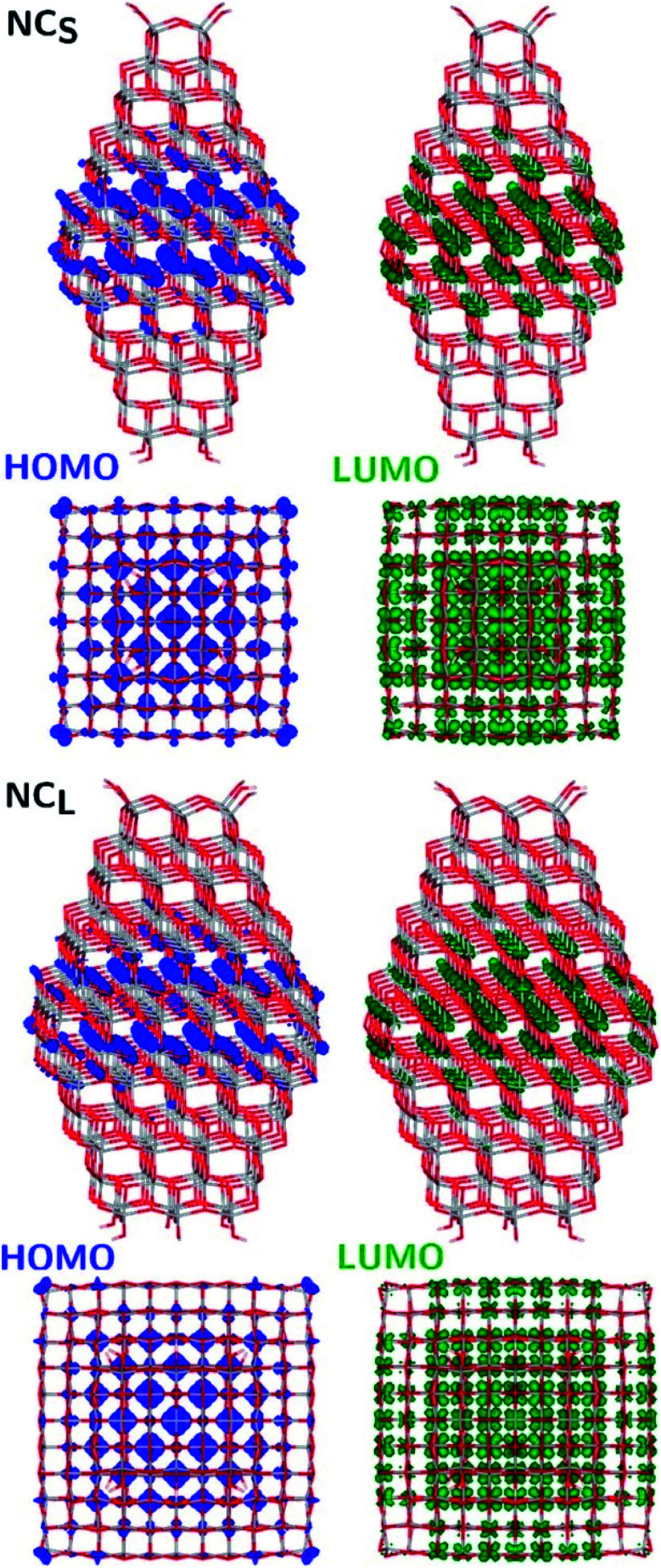
Side (upper) and top (lower) views of the electronic density plots of the frontier orbitals of NC_S_ and NC_L_ (isosurface = 5 × 10^−4^ au). Reprinted with permission from G. Fazio, L. Ferrighi and C. Di Valentin, *J. Phys. Chem. C*, 2015, **119**, 20735–20746. Copyright 2015 American Chemical Society.

**Fig. 11:**
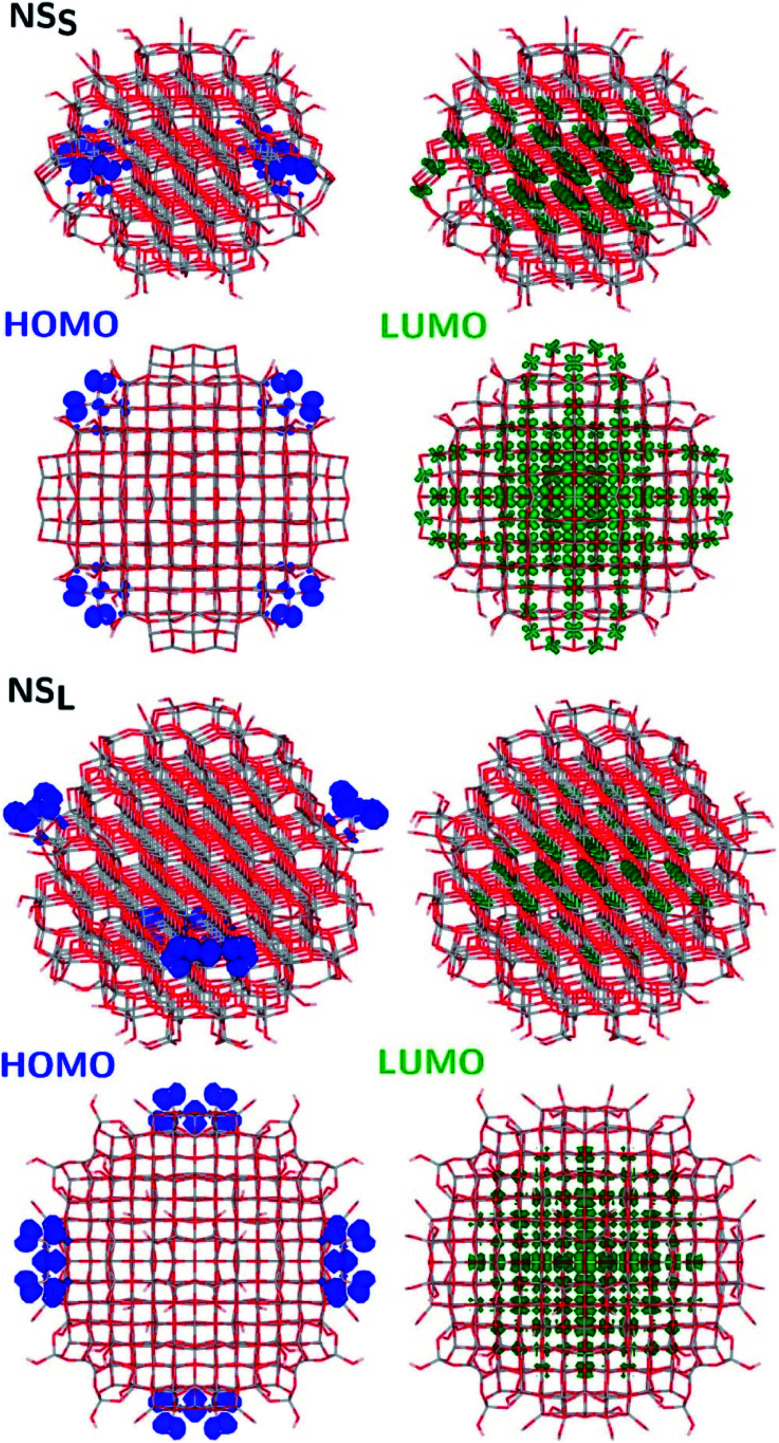
Side (upper) and top (lower) views of the electronic density plots of the frontier orbitals of NS_S_ and NS_L_ (isosurface = 5 × 10^−4^ au, except for HOMO, for which isosurface = 1 × 10^−3^ au). Reprinted with permission from G. Fazio, L. Ferrighi and C. Di Valentin, *J. Phys. Chem. C*, 2015, **119**, 20735–20746. Copyright 2015 American Chemical Society.

The structural differences between the faceted and spherical particles are reflected in marked differences in electronic properties. The authors compared the optical energy gap values, computed from the DFT Kohn–Sham orbital eigenvalues, and found that a sizable decrease is obtained going from the faceted to the spherical shape, in agreement with the work of Lamiel-Garcia *et al.* discussed above.^42^ The quantum confinement effect is also confirmed, since the optical energy gap value decreases with increasing size of the particle, for both spherical and faceted particles. Accordingly, larger NS particles show a lower optical energy gap. Analysis of the electron density distribution of the frontier molecular orbitals allows us to rationalize these trends; see Fig. 10. Indeed, the faceted particles show both HOMO and LUMO with electron density delocalized on the central belt of the particle. Conversely, the spherical particles show a HOMO with electron density fully localized on surface hydroxyls and a LUMO with electron density delocalized on the bulk Ti atoms (3d_*xy*_ states) rather than on the surface Ti atoms (Fig. 11). The different nature of the HOMO can be related to the smaller energy gap computed in spherical than in faceted particles and can induce a higher reductive power and a higher absorption capacity. The electronic structure calculations help to define the differences in the structural and electronic properties between the faceted and spherical nanoparticles, highlighting that faceted nanoparticles are more suited for applications where high crystallinity and low surface reactivity are fundamental, such as photovoltaics, while spherical nanoparticles are more suited for applications requiring high bonding energies for adsorbates, such as photocatalysis (Fig. 11).

The different nanoparticle morphologies also affect charge carrier transport through the mesoporous film. Storchi *et al.*^52^ carried out a computer simulation of the random packing of both rounded and faceted TiO_2_ nanoparticles, computing for the pyramidal particles a lower contact number, counting the number of adjacent overlapping nanoparticles and therefore pointing to the effective number of pathways through which the trapped electron can slip away on an adjacent particle. Accordingly, a more arduous percolation pathway of electrons has been envisaged in bipyramidal compared to spherical nanoparticles.

A more favorable situation for photocatalysis by rounded *vs.* faceted nanoparticles is also highlighted in the manuscript of Shirai *et al.*,^53^ where the effects of water molecules on the hole trapping mechanism at the surface of anatase TiO_2_ nanoparticles of different shapes have been investigated by DFT calculations. They demonstrated that surface OH groups on the curved surface of nanospheres effectively trap holes, unlike OH groups on the flat surface of faceted nanocrystals and this trapping ability is further enhanced by the adsorption of additional water molecules. This water-assisted effect is peculiar to the nanosphere morphology and originates from water adsorption as a ligand at low-coordinated Ti–OH sites or through robust hydrogen bonding directly to terminal OH at the highly curved surface. Remarkably, stronger hole trapping also enables a longer lifetime of electron carriers, so that, overall, the interaction of water molecules strengthens both the oxidative and the reductive power of the rounded TiO_2_ nanoparticles, while the faceted ones are almost unaffected.

The properties of the excited states of the TiO_2_ nanoparticles strongly affect photocatalytic activity.^54,55^ Regarding charge recombination dynamics, Nam *et al.*^56^ used non-adiabatic molecular dynamics calculations combined with real-time TDDFT to investigate the charge recombination rate in TiO_2_ nanoparticles of various sizes and shapes. They found that the charge recombination time increases with increasing particle size, as observed experimentally,^57^ due to the decrease in non-adiabatic coupling and exciton binding energy. In sufficiently large particles, the charge recombination dynamics also depend on the presence of defects, such as singly coordinated oxygen atoms or dopants (nitrogens or fluorines). The increase in the amount of defect sites increases the efficiency and duration of charge separation. Remarkably, photovoltaic applications require not only a long-lasting charge recombination time, but also efficient charge transport, which does not benefit from the presence of localized trap states originating from structural defects.

The nature of electronic trap states in realistic models of shape-tailored anatase TiO_2_ nanocrystals up to 6 nm in length has been investigated by quantum mechanical methods.^58,59^ The authors highlighted the presence of inherent trap states in perfectly stoichiometric and crystalline TiO_2_ bipyramidal nanocrystals, due to undercoordinated surface Ti(iv) ions at the (100) facets. The simulated adsorption of H_2_O molecules induces a reduction in localized trapped states at the bottom of the conduction band, while the sintering of two nanocrystals keeps the electron density distribution unaltered.^42^ Moving to more elongated, rod-shaped nanocrystals, showing a higher (100) to (101) surface ratio, a relatively deeper distribution of trap states has been pointed out, suggesting a potential enhancement in the photovoltaic performances of the material.^43^

## 3. Conclusions

To overcome the environmental and energy challenges that vex contemporary society, applications involving inorganic semiconductors, such as titanium dioxide, have gained much interest from the scientific community. Considerable progress has been attained in the last two decades with regard to the comprehension of the bulk and surface properties of TiO_2_; however, the characterization of nanostructured semiconductor materials, being highly problematic for both experimental and theoretical scientists, still involves extensive unexplored areas. The exploitation of anatase TiO_2_ nanoparticles in semiconductor photocatalysis requires intimate comprehension of the relationships between the structure and the optical properties affecting the photoactivity. In this respect, theoretical investigations based on quantum mechanical calculations can strongly support experimental outcomes. The present review reports the latest findings obtained from theoretical investigations of TiO_2_ nanoparticles, starting from an overview of the possible approaches to their modelling, with particular reference to reduced (1–3 nm diameter) and larger (>3 nm diameter) size particles. Through a bottom-up approach, reduced size particles can be simulated without the introduction of any idealization of the real particles, thus allowing the identification of various isomers differing in size, composition, morphology, and atomic and electronic structure. On the other hand, a top-down approach, generally involving cutting from a bulk material, is required for the modeling of more extended particles, so that a crucial feature for a reasonable description of nanoparticle properties is constituted by the adequate choice of the overall shape, exposed facets, and surface area of the particles. Scientific efforts point to the identification and rationalization of structural parameters that would allow an increase in the photocatalytic or photovoltaic efficiency of particles. It can be true that particles with a specific tailored shape perform better in photocatalytic applications than in photovoltaic ones. In general, a reduction in size is expected to enhance photoactivity, because of the higher surface-area ratio. However, for nanoparticles below 5 nm in diameter, a disruption in the crystalline order generally occurs, eventually involving the modification of the electronic properties with respect to those of bulk-like materials. The reduction in size also encompasses an increase in quantum confinement, which is related to an increase in the optical band gap, pointing to a decrease in the number of absorbed photons, but also to a decrease in the charge carrier recombination probability. Overall, prediction of the performance of a designed nanostructured material is quite challenging and, accordingly, this review discussed the main results relating to the dependence of photochemical properties on the size, morphology, and shape of TiO_2_ particles on the basis of quantum mechanical investigations.

For instance, the dependence of energetic stability on nanoparticle size was unraveled, focusing on the non-crystalline to crystalline crossover size, finding that nanocrystals with a diameter larger than 2–3 nm, owing to crystalline order, are energetically more stable than nanocrystals, owing to a disordered structure. This information is of fundamental importance for the adequate modeling of more extended nanoparticles. Then, the dependence of structural and electronic properties on nanoparticle size was considered, pointing to the range of sizes where these properties converge to those of the bulk materials. For *n* > 35, (TiO_2_)_*n*_ particles are found to retain an almost unaltered anatase structure. Since photocatalysis and photovoltaic applications strongly depend on the absorption properties of the material, the evaluation of the optical and electronic gaps and the excitation energy was discussed. Remarkably, the exciton binding energy, computed as the difference between the electronic and optical gaps, can provide an estimation of the difference in the electronic structure between nanoparticles and the bulk material. Octahedral nanoparticles with a diameter larger than 20 nm are predicted to exhibit a bulk-like electronic structure. By decreasing the size, the exciton binding energy is found to increase. Theoretical simulations also considered the effects of morphology, predicting a size range where spherical particles become energetically more stable than faceted ones upon an annealing process, keeping the crystalline order in the core and enforcing an amorphous structure in the outer part. Interestingly, spherical nanoparticles show an optical gap lower in energy than faceted ones, a feature which is particularly appealing for accomplishing an increase in the number of absorbed photons. These results suggest that band gap engineering can be obtained not only *via* controlling the size of the nanoparticles, but also *via* affecting their morphology.

The available theoretical studies on TiO_2_ nanostructures have allowed us to characterize the peculiar electronic and chemical properties in relation to the specific particle structure in terms of the size, shape, and morphology of the nanostructures. Nevertheless, further fundamental insights are highly desirable to further maximize the possible exploitation of semiconductor nanoparticles for improved technological applications.

## Author contributions

F. N. searched the literature, analyzed the results, and prepared the manuscript and figures. F. D. conceived the idea for the manuscript and supervised the work.

## Conflicts of interest

There are no conflicts to declare.

## Supplementary Material
